# Association of Trimethylamine *N*-Oxide and Metabolites With Mortality in Older Adults

**DOI:** 10.1001/jamanetworkopen.2022.13242

**Published:** 2022-05-20

**Authors:** Amanda M. Fretts, Stanley L. Hazen, Paul Jensen, Matthew Budoff, Colleen M. Sitlani, Meng Wang, Marcia C. de Oliveira Otto, Joseph A. DiDonato, Yujin Lee, Bruce M. Psaty, David S. Siscovick, Nona Sotoodehnia, W. H. Wilson Tang, Heidi Lai, Rozenn N. Lemaitre, Dariush Mozaffarian

**Affiliations:** 1 Department of Epidemiology, University of Washington School of Public Health, Seattle; 2 Cardiovascular Health Research Unit, University of Washington, Seattle; 3 Center for Microbiome and Human Health, Cleveland Clinic, Cleveland, Ohio; 4 Department of Cardiovascular Medicine, Heart, Vascular & Thoracic Institute, Cleveland Clinic, Cleveland, Ohio; 5 Department of Medicine, University of Washington, Seattle; 6 Lundquist Institute, Department of Medicine, Harbor UCLA Medical Center, Torrance, California; 7 Friedman School of Nutrition Science and Policy, Tufts University, Boston, Massachusetts; 8 Division of Epidemiology, Human Genetics and Environmental Sciences, University of Texas Health Science Center at Houston School of Public Health, Houston; 9 Department of Food and Nutrition, Myongji University, Yongin, Korea; 10 Department of Health Services, University of Washington, Seattle; 11 New York Academy of Medicine, New York City, New York; 12 Department of Primary Care and Public Health, Imperial College London, London, United Kingdom

## Abstract

**Importance:**

Little is known about the association of trimethylamine *N*-oxide (TMAO), a novel plasma metabolite derived from L-carnitine and phosphatidylcholine, and related metabolites (ie, choline, betaine, carnitine, and butyrobetaine) with risk of death among older adults in the general population.

**Objective:**

To investigate the associations of serial measures of plasma TMAO and related metabolites with risk of total and cause-specific death (ie, deaths from cardiovascular diseases [CVDs] and non-CVDs) among older adults in the US.

**Design, Setting, and Participants:**

This prospective cohort study involved 5333 participants from the Cardiovascular Health Study—a community-based longitudinal cohort of adults aged 65 years or older—who were followed up from June 1, 1989, to December 31, 2015. Participants were from 4 communities in the US (Forsyth County, North Carolina; Sacramento County, California; Washington County, Maryland; and Allegheny County, Pennsylvania). Data were analyzed from March 17 to June 23, 2021.

**Exposures:**

Plasma TMAO, choline, betaine, carnitine, and butyrobetaine levels were measured using stored samples from baseline (June 1, 1989, to May 31, 1990, or November 1, 1992, to June 31, 1993) and follow-up examination (June 1, 1996, to May 31, 1997). Measurements were performed through stable-isotope dilution liquid chromatography with tandem mass spectrometry using high-performance liquid chromatography with online electrospray ionization tandem mass spectrometry.

**Main Outcomes and Measures:**

Deaths (total and cause specific) were adjudicated by a centralized Cardiovascular Health Study events committee based on information from medical records, laboratory and diagnostic reports, death certificates, and/or interviews with next of kin. The associations of each metabolite with mortality were assessed using Cox proportional hazards regression models.

**Results:**

Among 5333 participants in the analytic sample, the mean (SD) age was 73 (6) years; 2149 participants (40.3%) were male, 3184 (59.7%) were female, 848 (15.9%) were African American, 4450 (83.4%) were White, and 35 (0.01%) were of other races (12 were American Indian or Alaska Native, 4 were Asian or Pacific Islander, and 19 were of other races or ethnicities). During a median follow-up of 13.2 years (range, 0-26.9 years), 4791 deaths occurred. After adjustment for potential confounders, the hazard ratios for death from any cause (ie, total mortality) comparing extreme quintiles (fifth vs first) of plasma concentrations were 1.30 (95% CI, 1.17-1.44) for TMAO, 1.19 (95% CI, 1.08-1.32) for choline, 1.26 (95% CI, 1.15-1.40) for carnitine, and 1.26 (95% CI, 1.13-1.40) for butyrobetaine. Plasma betaine was not associated with risk of death. The extent of risk estimates was similar for CVD and non-CVD mortality.

**Conclusions and Relevance:**

In this cohort study, plasma concentrations of TMAO and related metabolites were positively associated with risk of death. These findings suggest that circulating TMAO is an important novel risk factor associated with death among older adults.

## Key Points

**Question:**

Are trimethylamine *N*-oxide (TMAO), a novel plasma metabolite derived from L-carnitine and phosphatidylcholine, and related metabolites (ie, choline, betaine, carnitine, and butyrobetaine) associated with risk of death among older adults in the general US population?

**Findings:**

In this cohort study of 5333 older adults enrolled in the Cardiovascular Heart Study, TMAO and related metabolites were positively associated with risk of death, and this risk was highest among those with lower kidney function.

**Meaning:**

This study’s findings suggest that circulating TMAO is an important novel risk factor associated with risk of death among older adults.

## Teaser

This cohort study examines the associations of serial measures of plasma trimethylamine *N*-oxide and related metabolites with the risk of total and cause-specific death, including cardiovascular and noncardiovascular–related death, among older adults enrolled in the Cardiovascular Health Study.

## Introduction

There is increasing interest in the role of gut microbiota in health outcomes. In particular, high levels of trimethylamine *N*-oxide (TMAO), a novel plasma metabolite derived from L-carnitine and phosphatidylcholine (amino acids found in meats, eggs, and fish), have been positively associated with risk of death in several studies.^1,2,3,4,5,6,7,8,9,10,11,12,13,14,15,16,17,18^ To date, most studies that have assessed the association of circulating levels of TMAO with death in humans have focused primarily on populations with cardiometabolic diseases,^1,2,3,4,5,6,7,8,9,10,11,12,13,14,15,16,17,18^ including diabetes,^1^ chronic kidney disease,^2,3,4,5,6,7^ or cardiovascular disease (CVD).^8,9,10,11,12,13,14,15,16^ Because findings from studies involving populations with underlying morbidity may not be generalizable to healthy populations because of differences in health behaviors (eg, diet and medication use) and/or biological mechanisms underlying the association with TMAO levels, whether TMAO is associated with risk of death in the general population is unclear. Furthermore, most previous studies that have assessed associations between TMAO and health outcomes have focused solely on TMAO^1^ and used a single measure of TMAO at baseline.^2^ Because circulating levels of TMAO and related metabolites fluctuate over time, studies that use serial measures of the full range of TMAO and related metabolites and precursors are needed to better understand the long-term consequences of these metabolites for health.

The purpose of this cohort study was to investigate the associations of serial measures of plasma TMAO and its larger family of precursors (ie, choline, betaine, and carnitine) and metabolites (ie, butyrobetaine) with total and cause-specific mortality (ie, deaths from CVD and non–CVD-related causes) among older adults who participated in the Cardiovascular Health Study (CHS), a cohort study of risk factors associated with CVD among adults aged 65 years or older. We hypothesized that higher levels of TMAO and related precursors and metabolites would be associated with a higher risk of death in older adults.

## Methods

### Design and Population

The CHS was a community-based prospective cohort study of risk factors associated with CVD and stroke among older adults in the US. Details of the study design, sampling procedures, and data collection methods have been reported previously.^19^ In brief, the study used eligibility lists from the Centers for Medicare & Medicaid to recruit and enroll a random sample of noninstitutionalized adults aged 65 years or older from 4 communities (Forsyth County, North Carolina; Sacramento County, California; Washington County, Maryland; and Allegheny County, Pennsylvania). A total of 5201 participants were enrolled in the study from June 1, 1989, to May 31, 1990. An additional 687 participants (predominantly African American) enrolled in the study between November 1, 1992, and June 31, 1993. Participants completed annual clinic visits with interim phone calls every 6 months for the first 10 years of the study. Thereafter, participants were contacted every 6 months by phone for follow-up. Each center’s institutional review board approved the study protocol, and all participants provided written informed consent at enrollment. The current cohort study followed the Strengthening the Reporting of Observational Studies in Epidemiology (STROBE) reporting guideline for cohort studies.^20^

The current analytic sample comprised 5333 CHS participants with available TMAO measures from plasma samples at baseline (June 1, 1989, to May 31, 1990, or November 1, 1992, to June 31, 1993) and/or at follow-up examination (June 1, 1996, to May 31, 1997). Data were analyzed from March 17 to June 23, 2021. Because antibiotic use may affect the gut microbiota, we excluded 85 participants who reported any antibiotic use during the 2 weeks before blood sample collection.

### Data Collection

The CHS annual clinical examinations included standardized interviews, physical examinations, laboratory evaluations, and diagnostic testing as previously described.^19^ Information on medical history, educational level, smoking status, alcohol consumption, and CVD risk factors was collected as part of the standardized interviews. Dietary intake within the past year was measured using food frequency questionnaires administered in 1989 and 1996.^21,22^ Plasma blood samples were collected after a 12-hour overnight fast using ethylenediamine tetraacetic acid tubes and stored at −80 °C. Information on race and ethnicity was collected by participant self-report using prespecified categories: African American, American Indian or Alaska Native, Asian or Pacific Islander, White, and other race or ethnicity.

### Measurement of TMAO and Related Metabolites

Plasma TMAO, choline, betaine, carnitine, and butyrobetaine were measured at the Cleveland Clinic using stored samples from baseline (1989-1990 or 1992-1993) and follow-up examination (1996-1997). Measurements were performed with stable-isotope dilution liquid chromatography with tandem mass spectrometry using high-performance liquid chromatography with online electrospray ionization tandem mass spectrometry (4000 QTRAP LC-MS/MS System; SCIEX) and corresponding internal standard of d9(trimethyl)TMAO mass spectrometry (LCMS-8050 Triple Quadrupole Liquid Chromatograph Mass Spectrometer; Shimadzu Corporation).^23^ Concentrations of TMAO and d9(trimethyl)TMAO were monitored using electrospray ionization in positive ion mode with multiple reaction monitoring of parent ion transition m/z 76→58 and characteristic daughter ion transition m/z 85→66. Laboratory coefficients of variation varied by batch throughout the study period and by metabolite; the maximum coefficients of variation observed were 5.8% for TMAO, 4.1% for choline, 5.7% for carnitine, 7.0% for betaine, and 7.9% for butyrobetaine concentrations.

### Mortality Assessment

Deaths were adjudicated by a centralized CHS events committee based on information from medical records, laboratory and diagnostic reports, death certificates, and/or interviews with next of kin. Details of CHS methods for surveillance and disease classification have been reported in detail.^19,24^ For the purposes of this analysis, we evaluated the associations of concentrations of TMAO, choline, betaine, carnitine, and butyrobetaine with total mortality (primary outcome), CVD mortality (secondary outcome), and non-CVD mortality (ie, deaths from cancer, dementia, infection, respiratory diseases, or trauma or fracture; tertiary outcome). Study participants were followed up through 2015, for a maximum of 26 years.

### Statistical Analysis

For the primary analysis, each metabolite was assessed using quintiles, comparing each of the upper 4 quintiles with the lowest quintile (reference). For the supplementary analyses, linear assessments were performed for each metabolite after log-2 transformation. Test for trend was performed using a model that included the quintile variable as a linear term. Associations of each metabolite with total mortality (primary outcome), CVD mortality (secondary outcome), and non-CVD mortality (tertiary outcome) were assessed using Cox proportional hazards regression analysis. Participants were followed up from the time of their first metabolite measurement without concurrent antibiotic use until death or unavailability for follow-up through December 31, 2015.

Because we were most interested in evaluating long-term circulating metabolite levels, models included metabolite levels from their baseline measurement as the exposure until their second metabolite measurement, after which the cumulative mean of the 2 measurements was used. If a participant had only 1 metabolite measurement, that value was used as the exposure throughout the entire follow-up period for that participant. Overall, 2832 participants (53.1%) had 2 available or eligible metabolite measurements from baseline (1989-1990 or 1992-1993) and follow-up examination (1996-1997), 2051 participants (38.5%) had available or eligible metabolite measurements from baseline only, and 450 participants (8.4%) had available or eligible metabolite measurements from follow-up examination only and contributed to the analysis from the time of that measurement to December 31, 2015.

Quintile cutoffs for each metabolite were derived from the distribution of baseline measurements among the included population. For TMAO, quintile 1 was defined as 0.01 to 3.0 μm, quintile 2 as 3.1 to 4.1 μm, quintile 3 as 4.2 to 5.8 μm, quintile 4 as 5.9 to 9.1 μm, and quintile 5 as 9.2 to 255.0 μm. For choline, quintile 1 was defined as 0.2 to 7.7 μm, quintile 2 as 7.8 to 8.9 μm, quintile 3 as 9.0 to 10.1 μm, quintile 4 as 10.2 to 11.7 μm, and quintile 5 as 11.8 to 111.1 μm. For betaine, quintile 1 was defined as 0.4 to 27.1 μm, quintile 2 as 27.2 to 33.1 μm, quintile 3 as 33.2 to 38.9 μm, quintile 4 as 39.0 to 46.6 μm, and quintile 5 as 46.7 to 167.6 μm. For carnitine, quintile 1 was defined as 1.2 to 30.7 μm, quintile 2 as 30.8 to 34.9 μm, quintile 3 as 35.0 to 39.0 μm, quintile 4 as 39.1 to 43.9 μm, and quintile 5 as 44.0 to 95.0 μm. For butyrobetaine, quintile 1 was defined as 0.01 to 0.8 μm, quintile 2 as 0.9 to 0.98 μm, quintile 3 as 1.0 to 1.1 μm, quintile 4 as 1.2 to 1.3 μm, and quintile 5 as 1.4 to 4.6 μm.

Two levels of adjustment were used to examine the associations of TMAO, choline, betaine, carnitine, and butyrobetaine with mortality, with the adjustment covariates assessed at the time of each exposure measurement. For diet covariates, we created a cumulative mean for each food or nutrient in the analyses to reflect habitual diet; means were created based on responses to food frequency questionnaires administered in 1989 and 1996.^21,22^ The first model (minimally adjusted) included age, sex, race and ethnicity (African American vs other races or ethnicities), and enrollment site. Race and ethnicity was included as a sociocultural construct for confounding in analyses, reflecting historical and ongoing inequities that in part explain the high risk of death, particularly CVD death, among African American individuals.^25,26^ Because fewer than 1% of study participants reported racial or ethnic ancestry other than African American or White, we collapsed the race and ethnicity categories for analyses (ie, African American vs other races or ethnicities).

The second model (primary) also included educational level, household income, smoking status, body mass index (BMI; calculated as weight in kilograms divided by height in meters squared), physical activity, treated hypertension, instrumental activities of daily living, self-reported health status, systolic blood pressure, high-density lipoprotein cholesterol level, prevalent atrial fibrillation, prevalent coronary heart disease, history of myocardial infarction, prevalent diabetes, prevalent chronic obstructive pulmonary disease, and reported daily intake of eggs, fish, liver, nonprocessed red meat, processed meat, and total calories. Because an experimental study reported that TMAO increases cystatin C levels and is associated with kidney dysfunction (a risk factor associated with death),^2^ and TMAO is also cleared by the kidneys, it was possible that kidney function may have either mediated or impacted the association of TMAO and related metabolites with mortality. We therefore adjusted for estimated glomerular filtration rate (eGFR) in an exploratory model to assess the ways in which kidney function may have modified the association of TMAO and its related metabolites with mortality.

We also examined whether the association of each metabolite of interest with total mortality differed by sex, age, BMI, prevalent CVD, enrollment site, or eGFR in sensitivity analyses. Likelihood ratio tests were used to evaluate the statistical significance of the multiplicative interaction term for each factor, with each metabolite modeled as a linear term.

Missing covariates (0.01%-19.50%, depending on covariate at baseline [1989-1990 or 1992-1993]; 1.70%-20.20%, depending on covariate at follow-up examination [1996-1997]) were imputed using data on age, sex, race, clinic, educational level, household income, self-reported health status, smoking status, alcohol consumption, physical activity, instrumental activities of daily living, BMI, waist circumference, treated hypertension, insulin or oral hypoglycemic use, C-reactive protein, and eGFR via single imputation. Analyses in the CHS found that single imputation methods provided results similar to multiple imputation methods.^27^

A Bonferroni correction was used to address multiple comparisons. The significance level of main effects tests was set as 2-tailed *P* = .01 (.05 divided by 5 based on 1 primary death outcome and 5 metabolites). The correction for multiple comparisons in the interaction analyses was based on 5 interaction variables and 1 primary death outcome (.05 divided by 5, for a significance threshold of 2-tailed *P* = .01). The Grambsch-Therneau test based on Schoenfeld residuals was used to evaluate the proportional hazards assumption of the primary model for each metabolite^28^; there was no evidence that the proportional hazards assumption was violated for any of the metabolites. All statistical analyses were conducted using Stata software, version 16.0 (StataCorp LLC).

## Results

Among 5333 total participants in the analytic sample, the mean (SD) age was 73 (6) years; 2149 participants (40.3%) were male, 3184 (59.7%) were female, 848 (15.9%) were African American, 4450 (83.4%) were White, and 35 (0.01%) were of other races (12 were American Indian or Alaska Native, 4 were Asian or Pacific Islander, and 19 were of other races or ethnicities). Baseline levels of plasma TMAO and related metabolites varied widely across participants (eg, 1989-1990: median, 4.86 μm [range, 0.01-255.00 μm] for TMAO, 9.58 μm [range, 0.24-111.10 μm] for choline, 36.20 μm [range, 0.41-167.60 μm] for betaine, 36.80 μm [range, 1.22-95.00 μm] for carnitine, and 0.99 μm [range, 0.01-4.61 μm] for butyrobetaine) (Table 1). Spearman correlations between each of the metabolites were moderate, with the highest correlations observed for butyrobetaine and betaine (*r* = 0.40) and butyrobetaine and carnitine (*r* = 0.40) (eTable 1 in the Supplement).

**Table 1.** TMAO and Related Metabolites Among Cardiovascular Health Study Participants at Baseline^a^

Metabolite	Median (range), μm	
	1989-1990	1992-1993
TMAO	4.86 (0.01-255.00)	4.92 (0.09-306.00)
Choline	9.58 (0.24-111.10)	10.30 (3.22-35.00)
Betaine	36.20 (0.41-167.60)	35.40 (6.69-190.00)
Carnitine	36.80 (1.22-95.00)	35.80 (8.11-278.00)
Butyrobetaine	0.99 (0.01-4.61)	0.96 (0.07-3.46)

Abbreviation: TMAO, trimethylamine *N*-oxide.

^a^ A total of 5333 participants were included in the analysis.

Baseline characteristics of study participants according to quintile of plasma TMAO at baseline are shown in Table 2. Participants with higher vs lower levels of TMAO were older (eg, quintile 5 vs quintile 1: mean [SD] age, 74 [6] years vs 72 [5] years) and more likely to be male (eg, quintile 5 vs quintile 1: 485 men [45.1%] vs 340 men [32.0%]) and White (eg, quintile 5 vs quintile 1: 930 participants [86.4%] vs 844 participants [79.4%]). In addition, participants with higher vs lower levels of TMAO had lower high-density lipoprotein cholesterol levels (eg, quintile 5 vs quintile 1: mean [SD], 53 [16] mg/dL vs 57 [16] mg/dL; to convert to mmol/L, multiply by 0.0259) and eGFR levels (eg, quintile 5 vs quintile 1: mean [SD], 60 [19] mL/min/1.73 m^2^ vs 77 [14] mL/min/1.73 m^2^) and were more likely to report a history of myocardial infarction (eg, quintile 5 vs quintile 1: 139 participants [12.9%] vs 100 participants [9.4%]), coronary heart disease (eg, quintile 5 vs quintile 1: 254 participants [23.6%] vs 186 participants [17.5%]), or diabetes (eg, quintile 5 vs quintile 1: 204 participants [19.0%] vs 129 participants [12.1%]). Baseline characteristics of study participants according to levels of choline, betaine, carnitine, and butyrobetaine are available in eTables 2 to 5 in the Supplement.

**Table 2.** Baseline Characteristics of Cardiovascular Health Study Participants According to Plasma TMAO Levels at Baseline^a^

Characteristic	No. (%)					
	Total	TMAO quintile^b^				
		1	2	3	4	5
Total participants, No.	5333	1063	1053	1060	1081	1076
TMAO level, mean (range), μm	4.86 (0.01-255.00)	2.23 (0.01-3.00)	3.56 (3.00-4.10)	4.89 (4.10-5.80)	7.16 (5.80-9.10)	20.00 (9.10-255.00)
Age, mean (SD), y	73 (6)	72 (5)	73 (5)	73 (6)	74 (6)	74 (6)
Sex						
Male	2149 (40.3)	340 (32.0)	398 (37.8)	443 (41.8)	483 (44.7)	485 (45.1)
Female	3184 (59.7)	723 (68.0)	655 (62.2)	617 (58.2)	598 (55.3)	591 (54.9)
Race						
African American	848 (15.9)	211 (19.8)	173 (16.4)	172 (16.2)	153 (14.2)	139 (12.9)
White	4450 (83.4)	844 (79.4)	873 (82.9)	881 (83.1)	922 (85.3)	930 (86.4)
Other^c^	35 (0.7)	8 (0.8)	7 (0.7)	7 (0.7)	6 (0.6)	7 (0.7)
Smoking status						
Former	2224 (41.7)	376 (35.4)	439 (41.7)	443 (41.8)	479 (44.3)	487 (45.3)
Current	635 (11.9)	131 (12.3)	140 (13.3)	102 (9.6)	140 (13.0)	122 (11.3)
BMI, mean (SD)	27 (5)	26 (5)	27 (5)	27 (5)	27 (5)	27 (5)
SBP, mean (SD), mm Hg	136 (22)	136 (21)	136 (22)	136 (21)	138 (22)	135 (22)
HDL cholesterol, mean (SD), mg/dL	54 (16)	57 (16)	56 (16)	54 (15)	53 (15)	53 (16)
History of MI	581 (10.9)	100 (9.4)	100 (9.5)	110 (10.4)	132 (12.2)	139 (12.9)
Prevalent CHD	1098 (20.6)	186 (17.5)	194 (18.4)	211 (19.9)	253 (23.4)	254 (23.6)
Treated hypertension	2600 (48.8)	432 (40.6)	462 (43.9)	522 (49.2)	589 (54.5)	595 (55.3)
Prevalent diabetes	817 (15.3)	129 (12.1)	131 (12.4)	163 (15.4)	190 (17.6)	204 (19.0)
eGFR, mean (SD), mL/min/1.73 m^2^	68 (17)	77 (14)	72 (14)	68 (15)	64 (16)	60 (19)
Nonprocessed meat intake, mean (SD), servings/d	0.45 (0.36)	0.44 (0.34)	0.44 (0.34)	0.45 (0.34)	0.50 (0.41)	0.48 (0.35)
Processed meat intake, mean (SD), servings/d	0.39 (0.40)	0.37 (0.40)	0.38 (0.42)	0.38 (0.37)	0.42 (0.46)	0.38 (0.41)

Abbreviations: BMI, body mass index (calculated as weight in kilograms divided by height in meters squared); CHD, coronary heart disease; eGFR, estimated glomerular filtration rate; HDL, high-density lipoprotein; MI, myocardial infarction; SBP, systolic blood pressure; TMAO, trimethylamine *N*-oxide.

SI conversion factor: To convert to mmol/L, multiply by 0.0259.

^a^ Baseline was from 1989 to 1990 or 1992 to 1993.

^b^ Quintile 1 indicates 0.01-3.0 μm; quintile 2, 3.1-4.1 μm; quintile 3, 4.2-5.8 μm; quintile 4, 5.9-9.1 μm; and quintile 5, 9.2-255.0 μm.

^c^ Other races include American Indian (n = 12) or Alaska Native, Asian or Pacific Islander (n = 4), and other (n = 19).

During a median follow-up of 13.2 years (range, 0-26.9 years; 72 756 person-years), 4791 deaths occurred. Plasma TMAO was positively associated with total mortality. In the primary model, comparison of plasma TMAO concentrations in quintile 5 vs quintile 1 revealed a hazard ratio (HR) of 1.30 (95% CI, 1.17-1.44; *P* < .001 for trend) for death from any cause (Table 3). Higher levels of most of the other TMAO-related metabolites (ie, choline, carnitine, and butryrobetaine) were also associated with a higher risk of death compared with lower levels. For example, comparison of quintile 5 with quintile 1 revealed HRs for total death of 1.19 (95% CI, 1.08-1.32) for choline, 1.26 (95% CI, 1.15-1.40) for carnitine, and 1.26 (95% CI, 1.13-1.40) for butyrobetaine (*P* < .001 for trend for all comparisons). Plasma betaine was not associated with risk of death after correction for multiple testing (eg, quintile 5 vs quintile 1: HR, 1.11; 95% CI, 1.00-1.23; *P* = .02 for trend). The extent of risk estimates was similar for CVD mortality (eTable 6 in the Supplement) and non-CVD mortality (eTable 7 in the Supplement).

**Table 3.** Total Risk of Death According to TMAO and Related Metabolites Among Cardiovascular Health Study Participants, 1989-2015

Metabolite by quintile^a^	Model 1^b^		Model 2^c^	
	HR (95% CI)	*P* value for trend	HR (95% CI)	*P* value for trend
TMAO				
1	1 [Reference]	<.001	1 [Reference]	<.001
2	1.04 (0.95-1.14)		1.05 (0.96-1.16)	
3	1.10 (1.01-1.21)		1.10 (1.00-1.20)	
4	1.16 (1.06-1.27)		1.12 (1.02-1.22)	
5	1.36 (1.24-1.51)		1.30 (1.17-1.44)	
Choline				
1	1 [Reference]	<.001	1 [Reference]	<.001
2	0.91 (0.83-1.01)		0.98 (0.89-1.08)	
3	0.90 (0.81-0.99)		0.89 (0.81-0.99)	
4	0.99 (0.90-1.08)		0.99 (0.89-1.10)	
5	1.22 (1.11-1.34)		1.19 (1.08-1.32)	
Betaine				
1	1 [Reference]	.17	1 [Reference]	.02
2	0.97 (0.89-1.06)		1.02 (0.93-1.12)	
3	1.04 (0.95-1.13)		1.05 (0.96-1.16)	
4	1.02 (0.93-1.13)		1.10 (0.99-1.21)	
5	1.05 (0.95-1.16)		1.11 (1.00-1.23)	
Carnitine				
1	1 [Reference]	<.001	1 [Reference]	<.001
2	0.96 (0.88-1.05)		0.99 (0.90-1.09)	
3	0.95 (0.87-1.03)		1.01 (0.92-1.10)	
4	0.99 (0.91-1.08)		1.00 (0.92-1.10)	
5	1.25 (1.13-1.37)		1.26 (1.15-1.40)	
Butyrobetaine				
1	1 [Reference]	.001	1 [Reference]	<.001
2	1.03 (0.95-1.13)		1.08 (0.99-1.19)	
3	1.06 (0.97-1.17)		1.14 (1.04-1.25)	
4	1.13 (1.03-1.24)		1.20 (1.09-1.32)	
5	1.21 (1.09-1.35)		1.26 (1.13-1.40)	

Abbreviations: HR, hazard ratio; TMAO, trimethylamine *N*-oxide.

^a^ For TMAO, quintile 1 indicates 0.01-3.0 μm; quintile 2, 3.1-4.1 μm; quintile 3, 4.2-5.8 μm; quintile 4, 5.9-9.1 μm; and quintile 5, 9.2-255.0 μm. For choline, quintile 1 indicates 0.2-7.7 μm; quintile 2, 7.8-8.9 μm; quintile 3, 9.0-10.1 μm; quintile 4, 10.2-11.7 μm; and quintile 5, 11.8-111.1 μm. For betaine, quintile 1 indicates 0.4-27.1 μm; quintile 2, 27.2-33.1 μm; quintile 3, 33.2-38.9 μm; quintile 4, 39.0-46.6 μm; and quintile 5, 46.7-167.6 μm. For carnitine, quintile 1 indicates 1.2-30.7 μm; quintile 2, 30.8-34.9 μm; quintile 3, 35.0-39.0 μm; quintile 4, 39.1-43.9 μm; and quintile 5, 44.0-95.0 μm. For butyrobetaine, quintile 1 indicates 0.01-0.8 μm; quintile 2, 0.90-0.98 μm; quintile 3, 1.0-1.1 μm; quintile 4, 1.2-1.3 μm; and quintile 5, 1.4-4.6 μm.

^b^ Model 1 (minimally adjusted model) was adjusted for age, sex, race and ethnicity (African American vs other races or ethnicities), and enrollment site. Because less than 1% of study participants reported racial ancestry other than African American and White, racial categories were collapsed for the analyses (ie, African American vs other races).

^c^ Model 2 (primary model) was adjusted for all variables in model 1 plus educational level, household income, smoking status, body mass index, physical activity, treated hypertension, instrumental activities of daily living, self-reported health status, systolic blood pressure, high-density lipoprotein cholesterol, prevalent atrial fibrillation, prevalent coronary heart disease, history of myocardial infarction, prevalent diabetes, prevalent chronic obstructive pulmonary disease, and reported daily intake of eggs, fish, liver, nonprocessed red meat, processed meat, and total calories.

In exploratory analyses, the risk of death associated with all metabolites was attenuated when the primary model was also adjusted for eGFR. Comparing quintile 5 with quintile 1, the HRs for total mortality were 1.07 (95% CI, 0.96-1.19; *P* = .51 for trend) for plasma TMAO, 0.98 (95% CI, 0.88-1.09; *P* = .61 for trend) for choline, 1.08 (95% CI, 0.97-1.19; *P* = .07 for trend) for betaine, 1.16 (95% CI, 1.05-1.28; *P* = .02 for trend) for carnitine, and 0.95 (95% CI, 0.85-1.07; *P* = .45 for trend) for butyrobetaine (eTable 8 in the Supplement).

We found no significant evidence of interactions of TMAO and its metabolites with risk of death according to age, sex, BMI, enrollment site, or prevalent CVD. However, we observed evidence of an interaction of eGFR with TMAO (eg, eGFR of 30 mL/min/1.73 m^2^: HR, 1.14; 95% CI, 1.07-1.21; *P* < .001 for interaction), choline (eg, eGFR of 30 mL/min/1.73 m^2^: HR, 1.43; 95% CI, 1.23-1.67; *P* < .001 for interaction), and butyrobetaine (eg, eGFR of 30 mL/min/1.73 m^2^: HR, 1.19; 95% CI, 1.04-1.37; *P* < .001 for interaction) for risk of death (eTable 9 in the Supplement). Further analysis revealed that higher levels of these metabolites were associated with a higher risk of death among participants with low kidney function (eg, per 2-fold higher TMAO: HR, 1.14 [95% CI, 1.07-1.21] for eGFR of 30 mL/min/1.73 m^2^; HR, 0.87 [95% CI, 0.80-1.95] for eGFR of 105 mL/min/1.73 m^2^).

## Discussion

In this large prospective cohort study of older adults, higher levels of TMAO and related metabolites (ie, choline, carnitine, and butyrobetaine) were associated with an increased risk of death, particularly among individuals with low kidney function. These findings suggested that circulating TMAO is an important novel risk factor associated with death among older adults.

The findings expanded on those from previous studies^1,2,3,4,5,6,7,8,9,10,11,12,13,14,15,16,17,18,29,30,31,32,33^ that suggested an association of TMAO with mortality in clinically selected populations with underlying prevalent morbidity, such as CVD, diabetes, nonalcoholic fatty liver disease, chronic kidney disease, and hemodialysis. In those studies,^1,2,3,4,5,6,7,8,9,10,11,12,13,14,15,16,17,18,29,30,31,32,33^ the extent of the association of circulating TMAO with risk of death varied considerably; results from 3 meta-analyses^17,18,34^ revealed that individuals with the highest levels of circulating TMAO had a 47% to 91% higher risk of all-cause death compared with individuals with lower levels of circulating TMAO. Results from our population-based study suggested that individuals with higher levels of plasma TMAO had a 30% higher risk of death compared with individuals with lower levels. Differences in reported risk estimates may be due to underlying differences between study populations. In particular, it is unclear whether findings from clinical cohorts are generalizable to the general population because preexisting morbidity may impact health behaviors, such as diet, whereby the underlying disease has consequences for both the gut microbiome and the risk of death. Notably, our analysis expanded on previous work, suggesting that not only TMAO but its larger family of precursors (ie, choline, betaine, and carnitine) and metabolites (ie, butyrobetaine) are associated with risk of death.

Although the mechanisms by which TMAO is associated with mortality are complex and not fully elucidated, experimental studies have reported that high plasma levels of TMAO had adverse effects on multiple organ systems, including the heart, liver, and kidneys.^2,10,35,36,37,38^ In vitro and animal studies have found that TMAO also impacts cholesterol, sterol metabolism, and platelet hyperreactivity and promotes the development of atherosclerosis.^10,35,36,37^ High levels of TMAO promote oxidative stress and inflammation, potentially due to impairment of endothelial nitric oxide synthase activity.^38^ In the liver and intestines, TMAO inhibits bile acid synthesis.^35,37^

Trimethylamine *N*-oxide is also bidirectionally associated with kidney function because it is both cleared by the kidneys and, in experimental studies, has been associated with impaired kidney function and kidney fibrosis.^2,4,39^ Given kidney clearance of TMAO, its toxic effects could be more substantial in settings of reduced clearance. Higher levels of TMAO could also be a downstream consequence of reduced kidney function, producing confounding. Our findings suggested that the associations of TMAO and related metabolites with risk of death were at least partly attenuated after adjustment for eGFR. This result is consistent with an analysis of data from the Prevention of Renal and Vascular Endstage Disease (PREVEND) study,^39^ in which the observed multivariable-adjusted risk of all-cause mortality with TMAO comparing extreme quartiles (HR, 1.36; 95% CI, 0.97-1.91; *P* = .02 for trend) was attenuated after adjustment for urinary albumin excretion and eGFR (HR, 1.15; 95% CI, 0.81-1.64; *P* = .22 for trend). In that study,^39^ the association of TMAO with mortality was also modified by eGFR in unadjusted and age- and sex-adjusted analyses, with higher risk observed only in participants with eGFR lower than 90 mL/min/1.73 m^2^. However, these previous findings^39^ were based on only 322 deaths (compared with 4791 deaths in the present study), limiting the statistical power to confirm this interaction in fully adjusted analyses. In addition, the study^39^ only assessed TMAO at baseline, potentially increasing misclassification over time; did not assess other TMAO-related metabolites; and recruited participants from a single Dutch city (vs recruitment of participants from 4 large US communities in the present study), potentially limiting generalizability.

In our exploratory analyses, we also identified an interaction with eGFR for TMAO and 2 of its related metabolites (choline and butyrobetaine) with risk of death, with higher risk observed among those with lower kidney function.^4,39^ Our findings from a well-characterized population-based cohort of US adults using serial measures of TMAO, its related metabolites, and multiple confounding variables support the need to understand the mechanisms underlying the associations between TMAO, its metabolites, kidney function, and risk of death. Our results also highlight the need to examine whether drug or diet interventions that target decreasing TMAO levels improve kidney function and/or risk of death among older adults. Because circulating TMAO and related metabolites are complex markers that reflect many factors, including diet, gut microbiome, and metabolism, more work is needed to understand the impact of health behaviors and risk factors for circulating TMAO levels.

### Strengths and Limitations

This study has several strengths. The CHS was a large population-based cohort study of risk factors associated with CVD in adults aged 65 years or older, a population with a high risk of death. The prospective design and randomized sampling technique (based on eligibility lists from the Centers for Medicare & Medicaid) used for recruitment minimized the potential for both selection and recall biases. The use of standardized instruments to collect data on demographic characteristics, clinical risk factors, and lifestyle habits maximized our ability to adjust for confounding. In addition, TMAO and related metabolites were measured at 2 points, allowing us to account for changes in plasma levels of these metabolites over time.

The study also has limitations. Although we adjusted for several major risk factors associated with mortality, residual confounding and bias from unknown or inadequately measured factors is possible. The CHS comprised participants aged 65 years or older, and results may not be generalizable to younger adults. Although previous work has reported long-term stability of TMAO over 14 years of storage at −80 °C (*r*^2^ = 0.98) and over multiple freeze-thaw cycles (intercycle coefficient of variation <10%),^23^ the stability of the metabolite beyond 14 years is unknown.

## Conclusions

The findings of this cohort study suggested that higher concentrations of TMAO and related metabolites were associated with a higher risk of death among older adults. These risks were most substantial among those with lower kidney function.

## Notes

Supplement. **eTable 1.** Spearman Correlations of TMAO, Related Metabolites, and Covariates of Interest Among Cardiovascular Health Study Participants at Baseline (1989-1990 or 1992-1993) **eTable 2.** Baseline Characteristics of Cardiovascular Health Study Participants According to Plasma Betaine Levels at Baseline (1989-1990 or 1992-1993; N = 5333) **eTable 3.** Baseline Characteristics of Cardiovascular Health Study Participants According to Plasma Choline Levels at Baseline (1989-1990 or 1992-1993; N = 5333) **eTable 4.** Baseline Characteristics of Cardiovascular Health Study Participants According to Plasma Carnitine Levels at Baseline (1989-1990 or 1992-1993; N = 5333) **eTable 5.** Baseline Characteristics of Cardiovascular Health Study Participants According to Plasma Butyrobetaine Levels at Baseline (1989-1990 or 1992-1993; N = 5333) **eTable 6.** Hazard Ratios for Cardiovascular Mortality According to TMAO or Related Metabolites Among Cardiovascular Health Study Participants (1989-2015) **eTable 7.** Hazard Ratios for Noncardiovascular Mortality According to TMAO or Related Metabolites Among Cardiovascular Health Study Participants (1989-2015) **eTable 8.** Hazard Ratios for Total Mortality According to TMAO or Related Metabolites Among Cardiovascular Health Study Participants (1989-2015) **eTable 9.** Hazard Ratios for Total Mortality According to TMAO or Related Metabolites According to eGFR Levels Among Cardiovascular Health Study Participants (1989-2015)
