# Supplementary material for: Association of Trimethylamine N-Oxide and Metabolites With Mortality in Older Adults
Source: JAMA Netw Open. 2022 May 20;5(5):e2213242. doi: 10.1001/jamanetworkopen.2022.13242 (PMC9123496; doi:10.1001/jamanetworkopen.2022.13242)
Supplement: Supplement. — eTable 1. Spearman Correlations of TMAO, Related Metabolites, and Covariates of Interest Among Cardiovascular Health Study Participants at Baseline (1989-1990 or 1992-1993) eTable 2. Baseline Characteristics of Cardiovascular Health Study Participants According to Plasma Betaine Levels at Baseline (1989-1990 or 1992-1993; N = 5333) eTable 3. Baseline Characteristics of Cardiovascular Health Study Participants According to Plasma Choline Levels at Baseline (1989-1990 or 1992-1993; N = 5333) eTable 4. Baseline Characteristics of Cardiovascular Health Study Participants According to Plasma Carnitine Levels at Baseline (1989-1990 or 1992-1993; N = 5333) eTable 5. Baseline Characteristics of Cardiovascular Health Study Participants According to Plasma Butyrobetaine Levels at Baseline (1989-1990 or 1992-1993; N = 5333) eTable 6. Hazard Ratios for Cardiovascular Mortality According to TMAO or Related Metabolites Among Cardiovascular Health Study Participants (1989-2015) eTable 7. Hazard Ratios for Noncardiovascular Mortality According to TMAO or Related Metabolites Among Cardiovascular Health Study Participants (1989-2015) eTable 8. Hazard Ratios for Total Mortality According to TMAO or Related Metabolites Among Cardiovascular Health Study Participants (1989-2015) eTable 9. Hazard Ratios for Total Mortality According to TMAO or Related Metabolites According to eGFR Levels Among Cardiovascular Health Study Participants (1989-2015) [file jamanetwopen-e2213242-s001.pdf]

Supplementary Online Content

Fretts AM, Hazen SL, Jensen P, et al. Association of trimethylamine *N*-oxide and metabolites with mortality in older adults. *JAMA Netw Open*. 2022;5(5):e2213242. doi:10.1001/jamanetworkopen.2022.13242

eTable 1. Spearman Correlations of TMAO, Related Metabolites, and Covariates of Interest Among Cardiovascular Health Study Participants at Baseline (1989-1990 or 1992-1993)

eTable 2. Baseline Characteristics of Cardiovascular Health Study Participants According to Plasma Betaine Levels at Baseline (1989-1990 or 1992-1993; N = 5333)

eTable 3. Baseline Characteristics of Cardiovascular Health Study Participants According to Plasma Choline Levels at Baseline (1989-1990 or 1992-1993; N = 5333)

eTable 4. Baseline Characteristics of Cardiovascular Health Study Participants According to Plasma Carnitine Levels at Baseline (1989-1990 or 1992-1993; N = 5333)

eTable 5. Baseline Characteristics of Cardiovascular Health Study Participants According to Plasma Butyrobetaine Levels at Baseline (1989-1990 or 1992-1993; N = 5333)

eTable 6. Hazard Ratios for Cardiovascular Mortality According to TMAO or Related Metabolites Among Cardiovascular Health Study Participants (1989-2015)

eTable 7. Hazard Ratios for Noncardiovascular Mortality According to TMAO or Related Metabolites Among Cardiovascular Health Study Participants (1989-2015)

eTable 8. Hazard Ratios for Total Mortality According to TMAO or Related Metabolites Among Cardiovascular Health Study Participants (1989-2015)

eTable 9. Hazard Ratios for Total Mortality According to TMAO or Related Metabolites According to eGFR Levels Among Cardiovascular Health Study Participants (1989-2015)

This supplementary material has been provided by the authors to give readers additional information about their work.

© 2022 Fretts AM et al. *JAMA Network Open*.

eTable 1. Spearman Correlations of TMAO, Related Metabolites, and Covariates of Interest Among Cardiovascular Health Study Participants at Baseline (1989-1990 or 1992-1993)

	TMAO	Betaine	Choline	Carnitine	Butyrobetaine	Age	Sex	Race	Education	Physical Activity
**TMAO**	1.00									
**Betaine**	0.06	1.00								
**Choline**	0.22	0.38	1.00							
**Carnitine**	0.17	0.27	0.26	1.00						
**Butyrobetaine**	0.27	0.40	0.34	0.40	1.00					
**Age**	0.09	0.08	0.17	-0.02	0.09	1.00				
**Sex**	0.10	0.31	0.23	0.09	0.44	0.09	1.00			
**Race**	-0.06	0.05	-0.08	0.00	0.01	0.00	-0.04	1.00		
**Education**	0.02	0.02	-0.02	-0.05	0.00	-0.02	0.08	-0.12	1.00	
**Physical Activity**	-0.02	0.10	0.04	0.01	0.12	-0.12	0.25	-0.15	0.13	1.00

Abbreviations: TMAO, trimethylamine-N-oxide

© 2022 Fretts AM et al. *JAMA Network Open*.

eTable 2. Baseline Characteristics of Cardiovascular Health Study Participants According to Plasma Betaine Levels at Baseline (1989-1990 or 1992-1993; N = 5333)[a]

	I	II	III	IV	V
N	N=1043	N=1071	N=1064	N=1064	N=1091
Mean	21.6	30.3	36.0	42.4	56.7
Range	0.40, 27.1	27.1, 33.1	33.1, 38.9	38.9, 46.6	46.6, 167.6
Age, years	72 (5)	73 (6)	73 (6)	73 (5)	74 (6)
Sex, %					
Male	17%	32%	42%	47%	62%
Female	83%	68%	58%	53%	38%
Race, %					
African American	14%	16%	16%	18%	19%
Other[b]	86%	84%	84%	82%	81%
Smoking, %					
Former	39%	40%	42%	42%	47%
Current	11%	13%	14%	11%	10%
BMI, kg/m^2	27 (5)	27 (5)	27 (5)	27 (5)	26 (4)
Systolic blood pressure, mmHg	137 (22)	137 (22)	136 (21)	136 (22)	135 (22)
HDL, mg/dL	58 (17)	54 (15)	53 (15)	54 (16)	53 (14)
Hx of MI, %	8%	10%	12%	12%	13%
Prevalent CHD, %	17%	18%	22%	22%	23%
Treated hypertension, %	48%	48%	49%	50%	48%
Prevalent Diabetes, %	20%	16%	15%	15%	12%
eGFR, mL/min/1.73m^2	70 (18)	69 (17)	68 (17)	68 (17)	66 (18)
Non-processed meat intake, servings/day	0.46 (0.38)	0.46 (0.34)	0.48 (0.36)	0.46 (0.34)	0.46 (0.37)
Processed meat intake, servings/day	0.35 (0.41)	0.38 (0.38)	0.40 (0.43)	0.39 (0.40)	0.42 (0.44)

[a]Data presented are mean(SD) or %; [b] Other includes American Indian/Alaska Native, Asian/Pacific Islander, and white. Abbreviations: BMI, body mass index; CHD, coronary heart disease; eGFR, estimated glomerular filtration rate; HDL, high density lipoprotein; Hx, history; MI, myocardial infarction

© 2022 Fretts AM et al. *JAMA Network Open*.

eTable 3. Baseline Characteristics of Cardiovascular Health Study Participants According to Plasma Choline Levels at Baseline (1989-1990 or 1992-1993; N = 5333)[a]

	I	II	III	IV	V
N	N=1023	N=1035	N=1061	N=1076	N=1138
Mean	6.74	8.35	9.49	10.8	14.1
Range	0.20, 7.70	7.70, 8.90	8.90, 10.1	10.1, 11.7	11.7, 111.0
Age, years	72 (5)	72 (5)	73 (6)	73 (6)	75 (6)
Sex, %					
Male	24%	33%	40%	46%	56%
Female	76%	67%	60%	54%	44%
Race, %					
African American	23%	17%	15%	14%	14%
Other[b]	77%	83%	85%	86%	86%
Smoking, %					
Former	40%	41%	40%	43%	44%
Current	15%	12%	13%	10%	10%
BMI, kg/m^2	26 (5)	27 (5)	27 (5)	27 (5)	27 (5)
Systolic blood pressure, mmHg	135 (21)	135 (21)	137 (22)	136 (22)	139 (22)
HDL, mg/dL	58 (17)	55 (16)	54 (15)	53 (14)	52 (16)
Hx of MI, %	8%	8%	11%	11%	16%
Prevalent CHD, %	16%	17%	20%	22%	28%
Treated hypertension, %	43%	44%	47%	50%	58%
Prevalent Diabetes, %	16%	13%	14%	16%	18%
eGFR, mL/min/1.73m^2	76 (15)	73 (15)	70 (15)	66 (15)	57 (18)
Non-processed meat intake, servings/day	0.44 (0.38)	0.46 (0.35)	0.47 (0.36)	0.46 (0.32)	0.49 (0.38)
Processed meat intake, servings/day	0.35 (0.41)	0.37 (0.41)	0.39 (0.39)	0.39 (0.41)	0.44 (0.43)

[a]Data presented are mean(SD) or %; [b] Other includes American Indian/Alaska Native, Asian/Pacific Islander, and white. Abbreviations: BMI, body mass index; CHD, coronary heart disease; eGFR, estimated glomerular filtration rate; HDL, high density lipoprotein; Hx, history; MI, myocardial infarction

© 2022 Fretts AM et al. *JAMA Network Open*.

eTable 4. Baseline Characteristics of Cardiovascular Health Study Participants According to Plasma Carnitine Levels at Baseline (1989-1990 or 1992-1993; N = 5333)[a]

	I	II	III	IV	V
N	N=1088	N=1070	N=1067	N=1049	N=1059
Mean	26.6	32.9	36.9	41.3	49.6
Range	1.20, 30.7	30.7, 34.9	34.9, 39.0	39.0, 43.9	44.0, 95.0
Age, years	72 (5)	72 (5)	73 (6)	73 (6)	75 (6)
Sex, %					
Male	34%	38%	41%	43%	46%
Female	66%	62%	59%	57%	54%
Race, %					
African American	16%	16%	18%	16%	17%
Other[b]	84%	84%	82%	84%	83%
Smoking, %					
Former	39%	44%	41%	40%	45%
Current	9%	10%	12%	15%	14%
BMI, kg/m^2	26 (5)	26 (5)	27 (5)	27 (5)	27 (5)
Systolic blood pressure, mmHg	138 (22)	136 (21)	137 (22)	136 (22)	136 (21)
HDL, mg/dL	57 (16)	56 (16)	54 (15)	53 (15)	52 (15)
Hx of MI, %	9%	10%	11%	12%	13%
Prevalent CHD, %	19%	19%	20%	22%	23%
Treated hypertension, %	42%	46%	49%	50%	57%
Prevalent Diabetes, %	20%	15%	13%	15%	14%
eGFR, mL/min/1.73m^2	71 (17)	71 (17)	68 (17)	67 (17)	63 (18)
Non-processed meat intake, servings/day	0.46 (0.36)	0.44 (0.38)	0.46 (0.35)	0.45 (0.34)	0.45 (0.32)
Processed meat intake, servings/day	0.39 (0.41)	0.37 (0.42)	0.38 (0.41)	0.37 (0.41)	0.4 (0.4)

[a]Data presented are mean(SD) or %; [b] Other includes American Indian/Alaska Native, Asian/Pacific Islander, and white. Abbreviations: BMI, body mass index; CHD, coronary heart disease; eGFR, estimated glomerular filtration rate; HDL, high density lipoprotein; Hx, history; MI, myocardial infarction

© 2022 Fretts AM et al. *JAMA Network Open*.

eTable 5. Baseline Characteristics of Cardiovascular Health Study Participants According to Plasma Butyrobetaine Levels at Baseline (1989-1990 or 1992-1993; N = 5333)[a]

	I	II	III	IV	V
N	N=1048	N=1074	N=1051	N=1077	N=1083
Mean	0.65	0.85	0.99	1.16	1.56
Range	0.01, 0.80	0.80, 0.90	0.90, 1.10	1.10, 1.30	1.30, 4.60
Age, years	72 (5)	73 (6)	73 (6)	73 (6)	74 (6)
Sex, %					
Male	11%	25%	38%	52%	74%
Female	89%	75%	62%	48%	26%
Race, %					
African American	16%	16%	16%	17%	17%
Other[b]	84%	84%	84%	83%	83%
Smoking, %					
Former	35%	37%	41%	45%	50%
Current	13%	12%	12%	12%	12%
BMI, kg/m^2	27 (5)	27 (5)	27 (5)	27 (5)	26 (4)
Systolic blood pressure, mmHg	137 (21)	136 (21)	136 (22)	136 (22)	136 (23)
HDL, mg/dL	59 (16)	57 (16)	55 (15)	53 (15)	50 (14)
Hx of MI, %	7%	8%	12%	12%	15%
Prevalent CHD, %	17%	18%	21%	22%	26%
Treated hypertension, %	46%	47%	49%	51%	51%
Prevalent Diabetes, %	17%	16%	16%	14%	13%
eGFR, mL/min/1.73m^2	77 (15)	72 (15)	69 (15)	65 (16)	59 (18)
Non-processed meat intake, servings/day	0.41 (0.32)	0.45 (0.37)	0.45 (0.34)	0.47 (0.35)	0.53 (0.39)
Processed meat intake, servings/day	0.32 (0.36)	0.33 (0.37)	0.39 (0.42)	0.41 (0.42)	0.48 (0.47)

[a]Data presented are mean(SD) or %; [b] Other includes American Indian/Alaska Native, Asian/Pacific Islander, and white. Abbreviations: BMI, body mass index; CHD, coronary heart disease; eGFR, estimated glomerular filtration rate; HDL, high density lipoprotein; Hx, history; MI, myocardial infarction

© 2022 Fretts AM et al. *JAMA Network Open*.

eTable 6. Hazard Ratios for Cardiovascular Mortality According to TMAO or Related Metabolites Among Cardiovascular Health Study Participants (1989-2015)

	Model 1[a]		Model 2[b]		Model 3[c]	
	**HR (95% CI)**	**p-trend**	**HR (95% CI)**	**p-trend**	**HR (95% CI)**	**p-trend**
**TMAO**						
Q1	1.00 (Ref)	<0.0001	1.00 (Ref)	<0.0001	1.00 (Ref)	0.65
Q2	1.06 (0.90, 1.25)		1.06 (0.90, 1.25)		1.01 (0.86, 1.20)	
Q3	1.28 (1.10, 1.49)		1.23 (1.05, 1.44)		1.11 (0.95, 1.30)	
Q4	1.25 (1.07, 1.46)		1.13 (0.96, 1.32)		0.96 (0.82, 1.14)	
Q5	1.50 (1.27, 1.77)		1.35 (1.14, 1.60)		1.09 (0.91, 1.30)	
**Choline**						
Q1	1.00 (Ref)	<0.0001	1.00 (Ref)	0.004	1.00 (Ref)	0.91
Q2	0.93 (0.78, 1.10)		0.97 (0.82, 1.15)		0.93 (0.79, 1.11)	
Q3	0.94 (0.80, 1.11)		0.89 (0.75, 1.05)		0.84 (0.70, 0.99)	
Q4	1.13 (0.96, 1.33)		1.05 (0.89, 1.24)		0.93 (0.79, 1.11)	
Q5	1.39 (1.19, 1.63)		1.21 (1.02, 1.42)		0.98 (0.82, 1.17)	
**Betaine**						
Q1	1.00 (Ref)	0.18	1.00 (Ref)	0.04	1.00 (Ref)	0.08
Q2	0.94 (0.81, 1.10)		0.99 (0.84, 1.16)		0.98 (0.84, 1.15)	
Q3	1.09 (0.94, 1.27)		1.09 (0.93, 1.28)		1.07 (0.92, 1.26)	
Q4	1.06 (0.90, 1.24)		1.13 (0.96, 1.33)		1.11 (0.94, 1.31)	
Q5	1.06 (0.90, 1.26)		1.14 (0.96, 1.35)		1.11 (0.94, 1.32)	
**Carnitine**						
Q1	1.00 (ref)	0.003	1.00 (Ref)	0.02	1.00 (Ref)	0.18
Q2	1.00 (0.86, 1.16)		1.03 (0.88, 1.20)		1.02 (0.88, 1.19)	
Q3	0.96 (0.83, 1.12)		1.01 (0.87, 1.18)		0.99 (0.85, 1.15)	
Q4	1.11 (0.96, 1.29)		1.10 (0.94, 1.28)		1.05 (0.90, 1.23)	
Q5	1.24 (1.06, 1.46)		1.22 (1.04, 1.44)		1.12 (0.95, 1.32)	
**Butyrobetaine**						
Q1	1.00 (Ref)	0.10	1.00 (Ref)	0.07	1.00 (Ref)	0.10
Q2	1.14 (0.98, 1.33)		1.19 (1.02, 1.39)		1.09 (0.94, 1.28)	
Q3	1.12 (0.96, 1.31)		1.19 (1.01, 1.39)		1.02 (0.87, 1.20)	
Q4	1.13 (0.96, 1.33)		1.17 (0.99, 1.39)		0.96 (0.81, 1.13)	
Q5	1.20 (1.00, 1.44)		1.24 (1.03, 1.49)		0.90 (0.74, 1.09)	

[a] Adjusted for age, sex, race, and enrollment site; [b] Additionally adjusted for education, household income, smoking, BMI, physical activity, treated hypertension, instrumental activities of daily living, self-reported health status, systolic blood pressure, HDL cholesterol, prevalent atrial fibrillation, prevalent coronary heart disease, myocardial infarction, prevalent diabetes, prevalent chronic obstructive pulmonary disease, and reported daily intake of eggs, fish, liver, non-processed red meat, processed meat and total calories; [c]Additionally adjused for eGFR; Abbreviations: CI, confidence interval; HR, hazard ratio; Q, quintile; TMAO, trimethylamine-N-oxide

© 2022 Fretts AM et al. *JAMA Network Open*.

eTable 7. Hazard Ratios for Noncardiovascular Mortality According to TMAO or Related Metabolites Among Cardiovascular Health Study Participants (1989-2015)

	Model 1[a]		Model 2[b]		Model 3[c]	
	**HR (95% CI)**	**p-trend**	**HR (95% CI)**	**p-trend**	**HR (95% CI)**	**p-trend**
**TMAO**						
Q1	1.00 (Ref)	<0.0001	1.00 (Ref)	0.0003	1.00 (Ref)	0.61
Q2	1.03 (0.92, 1.15)		1.04 (0.93, 1.18)		1.00 (0.89, 1.13)	
Q3	1.01 (0.90, 1.14)		1.02 (0.91, 1.15)		0.93 (0.83, 1.05)	
Q4	1.11 (0.99, 1.25)		1.11 (0.99, 1.25)		0.97 (0.86, 1.09)	
Q5	1.29 (1.14, 1.46)		1.27 (1.12, 1.44)		1.06 (0.93, 1.21)	
**Choline**						
Q1	1.00 (Ref)	0.02	1.00 (Ref)	0.01	1.00 (Ref)	0.49
Q2	0.90 (0.80, 1.02)		0.98 (0.87, 1.11)		0.95 (0.84, 1.07)	
Q3	0.87 (0.77, 0.99)		0.90 (0.79, 1.02)		0.85 (0.74, 0.96)	
Q4	0.91 (0.81, 1.03)		0.96 (0.84, 1.09)		0.86 (0.75, 0.98)	
Q5	1.13 (1.00, 1.28)		1.18 (1.04, 1.34)		0.98 (0.86, 1.12)	
**Betaine**						
Q1	1.00 (Ref)	0.47	1.00 (Ref)	0.19	1.00 (Ref)	0.39
Q2	0.99 (0.89, 1.11)		1.03 (0.92, 1.16)		1.03 (0.92, 1.15)	
Q3	1.01 (0.90, 1.13)		1.02 (0.91, 1.15)		1.00 (0.89, 1.12)	
Q4	1.01 (0.89, 1.13)		1.07 (0.94, 1.21)		1.05 (0.93, 1.19)	
Q5	1.04 (0.92, 1.18)		1.08 (0.95, 1.23)		1.05 (0.93, 1.20)	
**Carnitine**						
Q1	1.00 (ref)	0.006	1.00 (Ref)	0.001	1.00 (Ref)	0.06
Q2	0.94 (0.84, 1.05)		0.97 (0.86, 1.08)		0.96 (0.85, 1.07)	
Q3	0.94 (0.84, 1.05)		1.00 (0.90, 1.12)		0.98 (0.88, 1.10)	
Q4	0.92 (0.83, 1.03)		0.95 (0.85, 1.07)		0.92 (0.82, 1.03)	
Q5	1.25 (1.11, 1.40)		1.29 (1.14, 1.45)		1.19 (1.05, 1.34)	
**Butyrobetaine**						
Q1	1.00 (Ref)	<0.0001	1.00 (Ref)	<0.0001	1.00 (Ref)	0.77
Q2	0.98 (0.87, 1.09)		1.03 (0.91, 1.15)		0.96 (0.85, 1.08)	
Q3	1.04 (0.92, 1.16)		1.12 (0.99, 1.26)		0.99 (0.88, 1.12)	
Q4	1.13 (1.00, 1.27)		1.21 (1.07, 1.37)		1.02 (0.90, 1.16)	
Q5	1.22 (1.07, 1.40)		1.27 (1.11, 1.46)		0.99 (0.85, 1.15)	

[a] Adjusted for age, sex, race, and enrollment site; [b] Additionally adjusted for education, household income, smoking, BMI, physical activity, treated hypertension, instrumental activities of daily living, self-reported health status, systolic blood pressure, HDL cholesterol, prevalent atrial fibrillation, prevalent coronary heart disease, myocardial infarction, prevalent diabetes, prevalent chronic obstructive pulmonary disease, and reported daily intake of eggs, fish, liver, non-processed red meat, processed meat and total calories; [c]Additionally adjused for eGFR; Abbreviations: CI, confidence interval; HR, hazard ratio; Q, quintile; TMAO, trimethylamine-N-oxide

© 2022 Fretts AM et al. *JAMA Network Open*.

eTable 8. Hazard Ratios for Total Mortality According to TMAO or Related Metabolites Among Cardiovascular Health Study Participants (1989-2015)

	HR (95% CI)	p-trend
**TMAO**		
Q1	1.00 (Ref)	0.51
Q2	1.00 (0.91, 1.11)	
Q3	1.00 (0.91, 1.10)	
Q4	0.96 (0.88, 1.06)	
Q5	1.07 (0.96, 1.19)	
**Choline**		
Q1	1.00 (Ref)	0.61
Q2	0.94 (0.85, 1.04)	
Q3	0.84 (0.76, 0.93)	
Q4	0.88 (0.80, 0.98)	
Q5	0.98 (0.88, 1.09)	
**Betaine**		
Q1	1.00 (Ref)	0.07
Q2	1.01 (0.93, 1.11)	
Q3	1.03 (0.94, 1.13)	
Q4	1.08 (0.98, 1.19)	
Q5	1.08 (0.97, 1.19)	
**Carnitine**		
Q1	1.00 (Ref)	0.02
Q2	0.98 (0.90, 1.07)	
Q3	0.99 (0.90, 1.08)	
Q4	0.97 (0.88, 1.06)	
Q5	1.16 (1.05, 1.28)	
**Butyrobetaine**		
Q1	1.00 (Ref)	0.45
Q2	1.01 (0.92, 1.10)	
Q3	1.00 (0.91, 1.10)	
Q4	1.00 (0.90, 1.10)	
Q5	0.95 (0.85, 1.07)	

[a]Adjusted for age, sex, race, and enrollment site, education, household income, smoking, BMI, physical activity, treated hypertension, instrumental activities of daily living, self-reported health status, systolic blood pressure, HDL cholesterol, prevalent atrial fibrillation, prevalent coronary heart disease, myocardial infarction, prevalent diabetes, prevalent chronic obstructive pulmonary disease, reported daily intake of eggs, fish, liver, non-processed red meat, processed meat and total calories, and eGFR; Abbreviations: CI, confidence interval; HR, hazard ratio; Q, quintile; TMAO, trimethylamine-N-oxide

© 2022 Fretts AM et al. *JAMA Network Open*.

eTable 9. Hazard Ratios for Total Mortality According to TMAO or Related Metabolites According to eGFR Levels Among Cardiovascular Health Study Participants (1989-2015)[a]

	**TMAO**	**Choline**	**Betaine**	**Carnitine**	**Butyrobetaine**
**eGFR**	**HR (95% CI)**	**HR (95% CI)**	**HR (95% CI)**	**HR (95% CI)**	**HR (95% CI)**
**30 mL/min/1.73m^2**	1.14 (1.07, 1.21)	1.43 (1.23, 1.67)	1.08 (0.94, 1.23)	1.18 (0.98, 1.42)	1.19 (1.04, 1.37)
**45 mL/min/1.73m^2**	1.08 (1.04, 1.13)	1.22 (1.09, 1.36)	1.07 (0.98, 1.17)	1.14 (1.01, 1.30)	1.09 (0.98, 1.21)
**60 mL/min/1.73m^2**	1.02 (0.99, 1.06)	1.04 (0.95, 1.14)	1.07 (1.00, 1.14)	1.11 (1.01, 1.22)	0.99 (0.92, 1.08)
**75 mL/min/1.73m^2**	0.97 (0.93, 1.02)	0.89 (0.80, 0.99)	1.06 (0.98, 1.15)	1.08 (0.95, 1.22)	0.91 (0.83, 0.99)
**90 mL/min/1.73m^2**	0.92 (0.86, 0.98)	0.76 (0.65, 0.88)	1.06 (0.94, 1.18)	1.05 (0.88, 1.25)	0.83 (0.74, 0.93)
**105 mL/min/1.73m^2**	0.87 (0.80, 0.95)	0.64 (0.53, 0.78)	1.05 (0.90, 1.23)	1.02 (0.79, 1.30)	0.75 (0.65, 0.88)

[a] Hazard ratios per two-fold higher metabolite. Adjusted for age, sex, race, and enrollment site, education, household income, smoking, BMI, physical activity, treated hypertension, instrumental activities of daily living, self-reported health status, systolic blood pressure, HDL cholesterol, prevalent atrial fibrillation, prevalent coronary heart disease, myocardial infarction, prevalent diabetes, prevalent chronic obstructive pulmonary disease, and reported daily intake of eggs, fish, liver, non-processed red meat, processed meat and total calories; Abbreviations: CI, confidence interval; eGFR, estimated glomerular filtration rate; HR, hazard ratio; TMAO, trimethylamine-N-oxide

© 2022 Fretts AM et al. *JAMA Network Open*.
